# Effects of Isometric Plantar-Flexion on the Lower Limb Muscle and Lumbar Tissue Stiffness

**DOI:** 10.3389/fbioe.2021.810250

**Published:** 2022-02-11

**Authors:** Baizhen Chen, Shaoyang Cui, Mingzhu Xu, Zhijie Zhang, Chunlong Liu

**Affiliations:** ^1^ Clinical Medical College of Acupuncture Moxibustion and Rehabilitation, Guangzhou University of Chinese Medicine, Guangzhou, China; ^2^ The Second Affiliated Hospital, Guangzhou University of Chinese Medicine, Guangzhou, China; ^3^ Shenzhen Hospital, Guangzhou University of Chinese Medicine (Futian), Shenzhen, China; ^4^ Shenzhen Hospital, Southern Medical University, Shenzhen, China; ^5^ Luoyang Orthopedics Hospital of Henan Province, Luoyang, China

**Keywords:** thoracolumbar fascia, gastrocnemius, shear wave elastography, myofascial continuity, isometric plantar-flexion, tensegrity network

## Abstract

**Purpose:** This study investigated the effects of isometric plantar-flexion against different resistances on the thoracolumbar fascia (TLF), erector spinae (ES), and gastrocnemius stiffness by shear wave elastography (SWE). The purpose was to explore the interaction between the lower limb muscle and lumbar tissue in the myofascial tensegrity network.

**Methods:** Twenty healthy young female were recruited in this study. The stiffness of the TLF, ES, medial gastrocnemius (MG), and lateral gastrocnemius (LG) was measured by SWE under four isometric plantar-flexion resistance conditions. The resistance conditions involved 0% maximum voluntary isometric contraction (MVIC), 20% MVIC, 40% MVIC, and 60% MVIC.

**Results:** There was a strong correlation between the stiffness change of MG and that of TLF (*r* = 0.768–0.943, *p* < 0.001) and ES (*r* = 0.743–0.930, *p* < 0.001), while it was moderate to strong correlation between MG and that of LG (*r* = 0.588–0.800, *p* < 0.001). There was no significant difference in the stiffness between the nondominant and dominant sides of TLF and ES under the resting position (*p* > 0.05). The increase in stiffness of the TLF, ES, MG, and LG, with MVIC percentage (*p* < 0.05), and the stiffness of TLF and ES on the nondominant side is much higher than that on the dominant side.

**Conclusions:** Our data shows that isometric plantar-flexion has a significant effect on the stiffness of the lumbar soft tissue and gastrocnemius. The gastrocnemius has a strong correlation with the stiffness changes of TLF and ES, which provides preliminary evidence for exploring the myofascial tensegrity network between the dorsal side of the lower limb muscle and lumbar tissue.

## Introduction

The fascia is a connective tissue network that envelops and links the muscles, blood vessels, nerves, and viscera of the whole body (Wilke et al., 2016; Bordoni et al., 2019). Fascia is mainly responsible for transmitting and absorbing loads to connective tissues, depending on its viscoelasticity, thereby building an extensive tensegrity network linking the various human tissues (Huijing, 2009). In recent years, biomechanical and anatomical studies have found that the interactions among different tissues is realized through the connection of muscle and fascia—for example, pectoralis major, latissimus dorsi, and deltoid transfer interact through the brachial fascia, thereby playing a synergistic role in upper arm movement (Stecco et al., 2008); dorsiflexion of the ankle joint can cause the joint displacement of the gastrocnemius, semimembranosus, and quadriceps femoris (Huang et al., 2018; Wilke et al., 2020); the contraction of gluteus maximus (GMax) can affect the contralateral latissimus dorsi through the thoracolumbar fascia (TLF) and erector spinae (ES) (Carvalhais et al., 2013). These studies show that the different human tissues interact by the myofascial tensegrity network, which provides solid evidence for the existence of the tensegrity network. However, the *in vivo* behavior of the tensegrity network lacks specific numerical data, so it is necessary to conduct clinical human trials of tensegrity networks in human tissues.

Anatomical studies have demonstrated a structural continuity between the lumbosacral portion and the lower limb. The TLF radiates outward along the L3-S3 spinous process and connects with GMax (Barker et al., 2014). GMax is continuous with fascia lata, iliotibial tract, and lateral muscle septum (Stecco et al., 2013). The hamstring and gastrocnemius are connected through a deep fascia of popliteal fossa (Tuncay et al., 2007). These anatomical studies provide a substantial way for the interaction between the dorsal side of lower limb muscle and lumbar tissue. Considering the existence of anatomical continuity between the lumbosacral and lower limbs, the change in lower limb strength may have a long-distance effect on the lumbar tissue. The gastrocnemius includes the medial gastrocnemius (MG) and lateral gastrocnemius (LG), which together with the soleus form the triceps surae. The triceps surae make an important contribution to human walking, running, and jumping by controlling the plantar-flexion movement (Fukunaga et al., 2001; Weinfeld, 2014). Fukunaga *et al*. found that the triceps surae are the largest synergist among plantar-flexors (Fukunaga et al., 1992). During plantar-flexion, these three muscles produce tension and act on the Achilles tendon (Rosager et al., 2002). Creswell *et al*. found that the gastrocnemius produces had the highest percentage of plantar-flexion torque knee extension position and that plantar-flexion can maximize gastrocnemius activity (Cresswell et al., 1995). Similarly, isometric contraction can maximize muscle activity. Therefore, we chose isometric plantar flexion in the prone position to maximize gastrocnemius tension in order to better observe the effects of isometric plantar-flexion on the dorsal side of lower limb muscle and lumbar tissue stiffness.

In the human body, the fascia is an important buffer structure and force transmitter. Its function depends on its own tension element, and tissue stiffness is one of the important indexes to evaluate tissue tension (Swanson, 2013). Shear wave elastography (SWE) is a new evaluation technology of tissue stiffness, which has the characteristics of being noninvasive, painless, fast, and accurate. SWE quantifies tissue stiffness and draws a colored image of tissue stiffness using shear wave generated by acoustic radiation force pulse and ultra-high speed imaging technology (Creze et al., 2018; Gennisson et al., 2013). Our previous research shows that SWE is a reliable tool for quantifying TLF, MG, and LG stiffness and monitoring its dynamic changes (Chen et al., 2021; Liu et al., 2020). Therefore, we used SWE to quantify the stiffness of TLF, ES, and gastrocnemius during isometric plantar-flexion in this experiment.

The purpose of this study was to assess the variations in stiffness of MG, LG, TLF, and ES at isometric plantar-flexion against different resistances by using the SWE. We put forward the following assumptions around the purpose of the study: (1) the stiffness of nondominant and dominant lumbar tissues increased asymmetrically with the increase of isometric plantar-flexion resistance; (2) the stiffness of gastrocnemius increased with the increase of isometric plantar-flexion resistance, and the stiffness of MG and LG increased unevenly; and (3) there was a significant correlation between the stiffness changes of the lower leg and the back tissue.

## Methods

### Ethical Approval

This study received approval by the ethics committee of the Guangdong Provincial Hospital of Chinese Medicine (YE 2020-131-01). This research follows the principles of the Helsinki Declaration. Before the experiment, all the participants fully understood the safety of SWE, the basic rights of the participants, the experimental purpose, and the process by a written agreement, and they signed an informed consent.

### Participants

The researchers recruited 20 healthy young female participants from Guangzhou University of Chinese Medicine from November 2020 to March 2021 (mean age: 21.1 ± 1.7 years, 19–23 years; mean height: 1.61 ± 0.04 m, 1.53–1.69 m; mean mass: 49.5 ± 5.1 kg, 41–59 kg; MVIC: 31.08 ± 4.79 kg). The location of the study was the Department of Ultra-sound Imaging of the Second Affiliated Hospital of Guangzhou University of Chinese Medicine. The inclusion criteria were that (1) all participants had no pain or trauma to their feet, lower limbs, and waist that affected their lives and work for, at least, the past 6 months; (2) all the participants had no history of surgery on their feet, lower limbs, and waist and had no history of neuromuscular diseases and joint diseases; and (3) with the right hands and feet on the dominant side (the dominant leg of the participants was determined by kicking a ball, and the dominant hand was determined by writing preference). The participants were instructed to abstain from physical activity for 48 h before the start of the experiment.

### Equipment and Parameter Settings

The study was conducted using an Aixplorer ultrasound device (Aixplorer Supersonic Imagine, France). A 40-mm linear array sensor (SL10-2, Supersonic Imagine, France) is selected for the instrument that was a handheld device. Musculoskeletal mode and SWE mode were selected for device mode. The equipment parameter settings were as follows : conventional preset enhancement mode, image display of 85% opacity, and measurement range from 0 to 300 kPa. The ergometer is HOGGAN Scientific microFET2 (wireless manual muscle tester; manufactured and calibrated in the USA), which converts the pressure signal into pounds through the pressure sensor and displays it on the screen.

### Experimental Protocol

**FIGURE 1:**
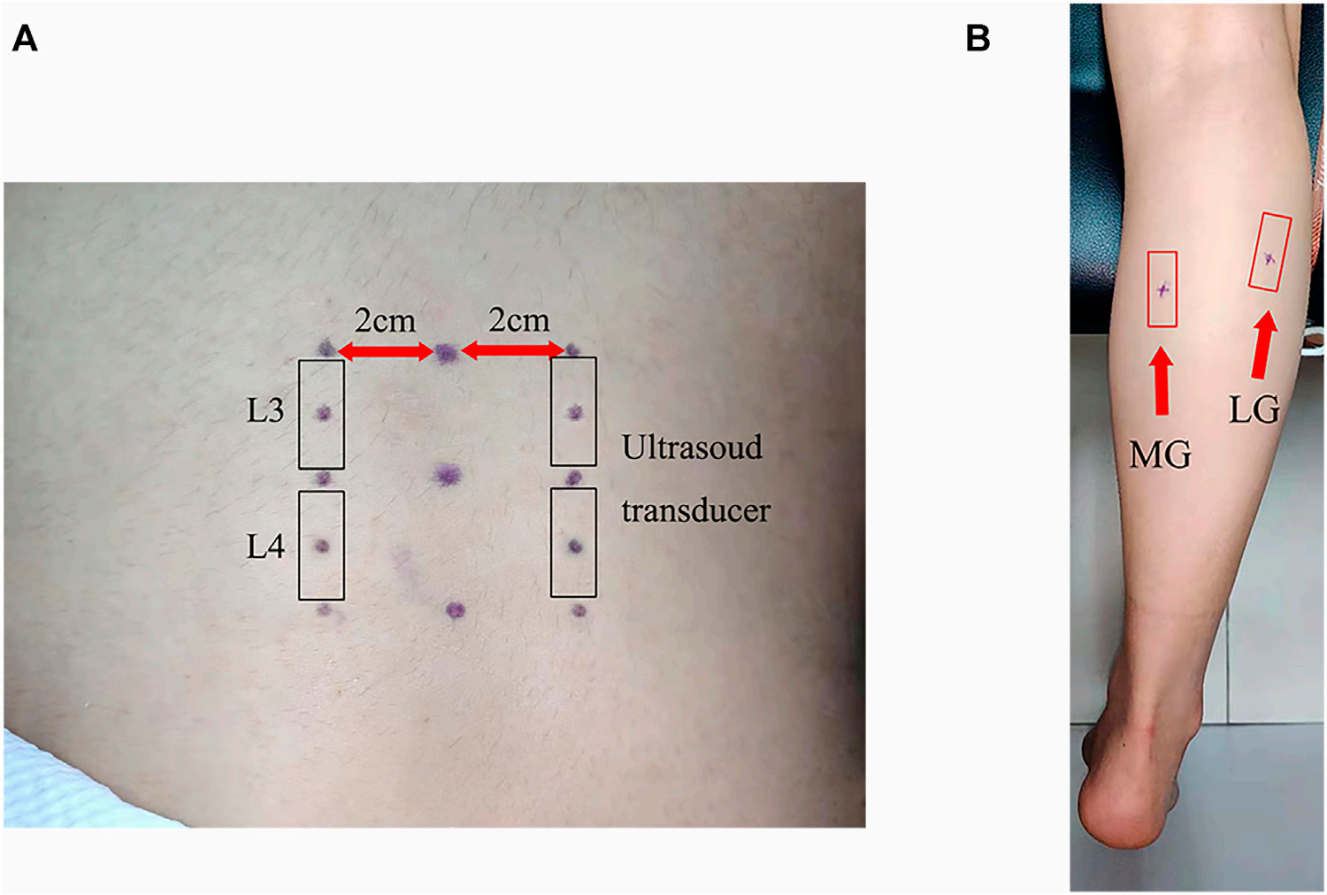
Setting out plan of the ultrasound transducer: **(A)** thoracolumbar fascia and erector spinae and **(B)** medial gastrocnemius and lateral gastrocnemius.

In this experiment, SWE was used to measure the stiffness of TLF, ES, MG, and LG. The lumbar tissue (TLF and ES) stiffness was measured at the third and fourth lumbar vertebra levels (L3 and L4). Firstly, the L4 spinous process was palpated according to the body surface markers, and the L3 spinous process and the L2 spinous process were palpated upward in turn. Then, B-mode ultrasound was used for confirmation and correction, and an oil pen was used to mark the measuring points 2 cm away from the midpoint of L2-3 and L3-4. The measurement site of MG was located at 30% of the length from the medial popliteal fossa to the lateral malleolus and that of LG was located at 30% of length from the lateral popliteal fossa to the medial malleolus. These measuring parts were marked with an oil pen (Figure 1).

**FIGURE 2:**
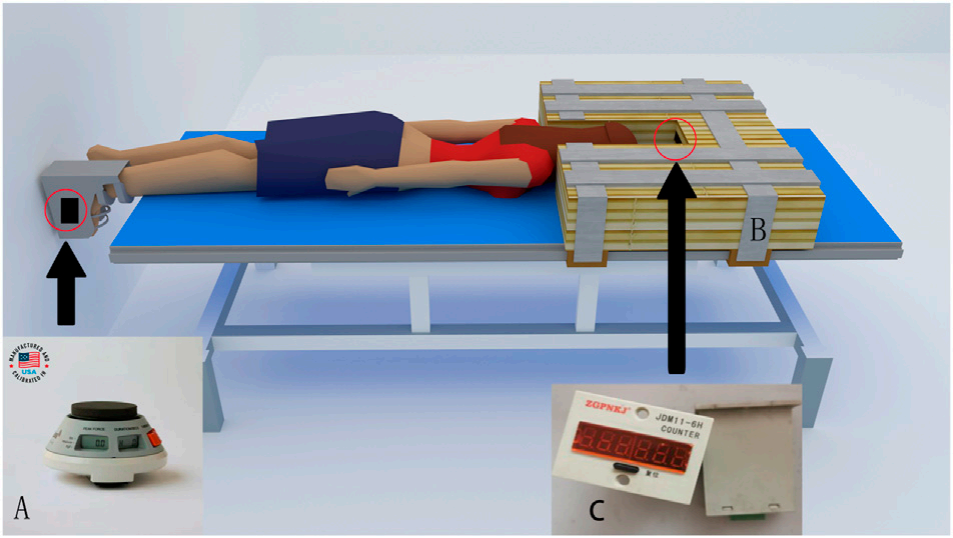
The posture and equipment used for measurement: **(A)** ergometer, **(B)** wooden block, and **(C)** monitor.

During the measurement period, all participants remained in the prone position with the hands at both sides of their body, the lower limbs straight, and the ankle joint at 90° position (Figure 2). After the participants were enrolled in the study, we conducted isometric plantar-flexion instruction for all participants. The participants were required to contract only the plantar-flexors (MG and LG) during isometric plantar-flexion and not the auxiliary muscle (hamstrings and GMax). The participants repeatedly practiced isometric plantar-flexion until they could easily use the plantar-flexors to complete the plantar-flexion.

The experiment was carried out according to the following steps: (1) the participants walked to the ultrasound room on the 4th floor of the hospital, they were fixed on a special bed according to the test requirements, their ankles were fixed at 90° with a special ankle foot orthosis (an ergometer at the bottom), their shoulder was fixed with a wooden block, and then they rested in the prone position for 5 min; (2) the stiffness of TLF, ES, MG, and LG was measured by SWE; (3) the participants were required to perform the maximum isometric plantar-flexion, and the ergometer value was recorded, which was the MVIC. The calculation of resistance value required for each contraction was as follows: 20% MVIC = 0.2 MVIC, 40% MVIC = 0.4 MVIC, and 60% MVIC = 0.6 MVIC. At each contraction, the participants were informed of the target resistance. The participants viewed the resistance value through the screen and kept the displayed value at the target value; (4) SWE was used to monitor the stiffness of the measured point until the stiffness value recovers to the value measured in step (2); (5) the participants were asked to perform 20% MVIC isometric plantar-flexion, and the stiffness of TLF, ES, MG, and LG were measured; (6) after repeating step (4), the participants were instructed to perform isometric plantar-flexion with 40% MVIC, and the stiffness of TLF, ES, MG, and LG was measured; and (7) after repeating step (4), the participants were ordered to perform 60% MVIC isometric plantar-flexion, and the stiffness of TLF, ES, MG, and LG was measured.

In the process of image acquisition, the B-mode was used to obtain clear images, and then the preset SWE mode was used to quantify the stiffness of each tissue. Each MVIC contraction was held for 8 s (5 s of stable image and elastic value and 3 s of intercepted image). The measurements were carried out in the order of MG, LG, TLF, and ES. One position was measured for each contraction, and between two contractions, SWE was used to monitor the stiffness of the measured point until the stiffness value recovered to the original stiffness (the value measured in step 2), then followed by the next contraction. Another researcher used the region of interest (ROI; system tool for automatically quantifying the shear modulus) of Aiexplorer software to obtain the shear modulus (kPa) from the intercepted image. The size of the ROIs of TLF was set to 1 mm, three adjacent ROIs were set for each measurement area to read data, and the size of ROI of ES, MG, and LG were set to 5 mm (Supplementary Figures S1-S3). Each measuring point is measured three times, and the average value was taken and used for further analyses.

### Statistical Analysis

**FIGURE 3:**
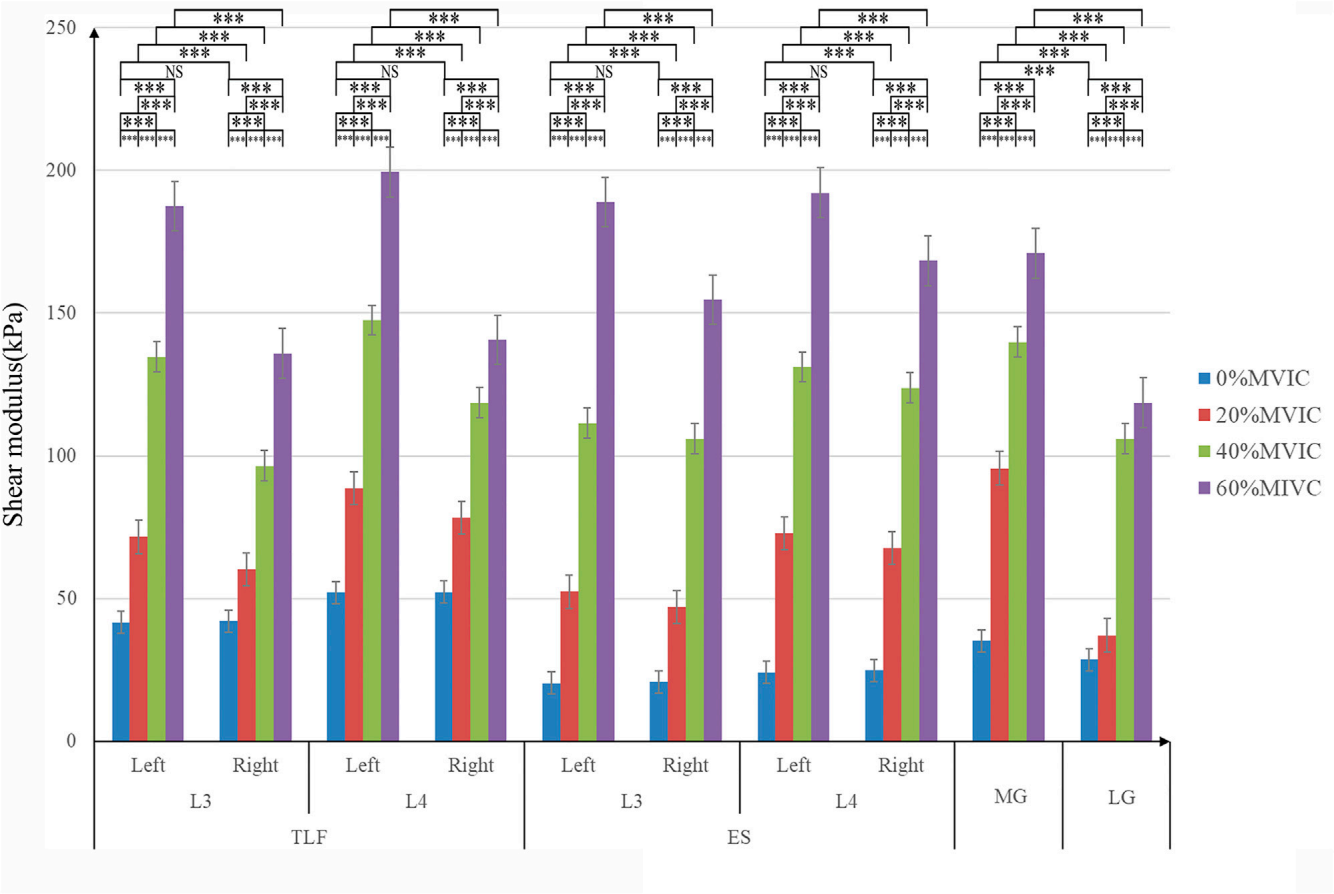
Stiffness variation of each tissue under different resistance conditions. ***, significant intergroup difference (*p* < 0.001); NS, non-significant intergroup difference (*p* > 0.05).

SPSS 21.0 software (version 21.0, Chicago, IL, USA) was used for statistical analysis. The statistical data was the average of the three measurements, and all statistical data were expressed as mean ± standard deviation (SD). The Shapiro–Wilk test was used for normal distribution of stiffness and stiffness percentage change data, and all data conformed to normal distribution. The one way-ANOVA with stiffness (the same measuring position) as the dependent variable and 0% MVIC, 20% MVIC, 40% MVIC, and 60%MVIC as the independent variables was used to judge whether there were differences in stiffness between different MVICs (Figure 3). When the one-way ANOVA was significant (Supplementary Table S1), *post-hoc* Tukey’s test was performed (Supplementary Table S2). The paired *t*-test was performed to verify the difference in lumbar tissue stiffness (the same tissue and lumbar vertebra level) between the dominant and nondominant sides (Figure 3). The significance level of the statistical data difference was set as *p* = 0.05.

Pearson’s correlation was used to calculate the correlation coefficients (*r*-values) of stiffness percentage change between different tissues. The raw values of stiffness reflected the absolute change of tissue under mechanical force loaded, in which it was easy to ignore the stiffness characteristics of the tissue itself. The stiffness percentage change was calculated by the ratio of absolute change of tissue stiffness with its initial stiffness, which truly reflected the influence of the mechanical force on the tissue itself. Therefore, we chose the stiffness percentage change of different tissues to calculate the correlation coefficients as follows: *Stiffness percentage change (%) = (measured stiffness − original stiffness) ÷ original stiffness* × 100% The stiffness percentage change of each measured position was compared to the stiffness percentage change of MG for the same test conditions. The correlation strength of the *r*-value was set to *r* <0.3 for weak correlation, 0.3 ≤ *r* ≤ 0.6 for moderate correlation, and *r* >0.6 for a strong correlation. The statistical significance level was set as *p* = 0.05.

## Results

### Effect of Different Isometric Plantar-Flexion Resistance on Tissue Stiffness

The relationship between tissue stiffness and different isometric plantar-flexion resistance is shown in Figure 3; Supplementary Figure S4. The stiffness of TLF, ES, MG, and LG increased significantly with the increase of resistance (*p* < 0.001, Figure 3). In the condition of no resistance, there was no significant difference in the stiffness between the nondominant and dominant sides of TLF and ES (*p* > 0.05, Figure 3), and MG stiffness was significantly higher than LG (*p* < 0.001, Figure 3). With the increase of resistance, the stiffness of nondominant TLF and ES was always greater than that of the dominant side (*p* < 0.001, Figure 3).

### Characteristics of Tissue-Stiffness-Phased Percentage Change Under Continuous Loading Resistance

The characteristics of tissue-stiffness-phased percentage change are shown in Table 1. The stiffness of ES and MG increased sharply when the resistance appears, and then the stiffness-phased percentage change decreased. The stiffness-phased percentage change of LG began to increase sharply at approximately 40% MVIC resistance. The phased percentage change of TLF stiffness increased continuously between 0 and 40% MVIC and decreased between 40 and 60% MVIC.

**TABLE 1:** Tissue stiffness phase percentage change (mean ± SD, %).

Measurement position			0–20% MVIC	20–40% MVIC	40–60% MVIC
TLF	L3	Left	73.49 ± 21.78	88.00 ± 4.55	39.43 ± 4.98
		Right	44.03 ± 24.83	60.75 ± 15.11	41.54 ± 9.97
	L4	Left	71.41 ± 19.96	66.74 ± 5.97	35.12 ± 3.92
		Right	50.67 ± 16.06	51.31 ± 8.35	19.18 ± 6.96
ES	L3	Left	160.01 ± 55.18	114.88 ± 17.28	69.78 ± 6.01
		Right	130.71 ± 52.98	128.65 ± 26.67	45.91 ± 5.89
	L4	Left	204.58 ± 50.99	81.00 ± 10.12	46.33 ± 7.51
		Right	174.64 ± 49.36	84.97 ± 17.24	36.47 ± 16.77
	MG	—	175.83 ± 52.76	46.87 ± 9.57	22.23 ± 6.26
	LG	—	31.34 ± 18.73	188.90 ± 39.92	12.63 ± 13.14

SD, standard deviation (kPa); TLF, thoracolumbar fascia; ES, erector spinae; L3, third lumbar vertebra levels; L4, fourth lumbar vertebra levels; MG, medial gastrocnemius; LG, lateral gastrocnemius.

### Correlation Between the Stiffness of Lumbar Tissue and Lower Limb Under Different Resistance

Each stiffness percentage change of every measurement position was compared with the stiffness percentage change of MG for the same test conditions (Table 2). The results showed that the stiffness percentage change of TLF (*r* = 0.768–0.936, *p* <0.001) and ES (*r* = 0.743–0.930, *p* < 0.001) has a strong correlation with the stiffness percentage change of MG under different resistance. The correlation of hardness percentage change between LG and MG was moderate and strong (*r* = 0.588–0.800, *p* < 0.001).

**TABLE 2:** The correlation between the stiffness percentage changes of each tissue (mean ± SD, %).

Measurement parts			0–20% MVIC			0–40% MVIC			0–60% MVIC		
			Percentage change	*r*-values	*p*-values	Percentage change	*r*-values	*p*-values	Percentage change	*r*-values	*p*-values
TLF	L3	Left	73.49 ± 21.78	0.936	0.000	226.69 ± 45.70	0.913	0.000	353.69 ± 51.29	0.847	0.000
		Right	44.03 ± 24.83	0.943	0.000	130.93 ± 40.91	0.898	0.000	225.36 ± 64.92	0.850	0.000
	L4	Left	71.41 ± 19.96	0.912	0.000	185.32 ± 30.53	0.927	0.000	285.75 ± 44.72	0.883	0.000
		Right	50.67 ± 16.06	0.839	0.000	128.31 ± 29.56	0.877	0.000	170.40 ± 23.20	0.768	0.000
ES	L3	Left	160.01 ± 55.18	0.910	0.000	450.89 ± 82.36	0.907	0.000	831.82 ± 116.02	0.858	0.000
		Right	130.71 ± 52.98	0.926	0.000	418.66 ± 94.99	0.889	0.000	656.62 ± 138.90	0.868	0.000
	L4	Left	204.58 ± 50.99	0.930	0.000	446.75 ± 65.19	0.925	0.000	702.95 ± 125.01	0.887	0.000
		Right	174.64 ± 49.36	0.884	0.000	401.15 ± 54.98	0.743	0.000	576.86 ± 44.97	0.809	0.000
	MG	—	175.83 ± 52.76	1	—	302.91 ± 66.83	1	—	392.95 ± 91.29	1	—
	LG	—	31.34 ± 18.73	0.800	0.000	276.53 ± 54.80	0.703	0.001	320.53 ± 57.57	0.588	0.006

SD, standard deviation (kPa); TLF, thoracolumbar fascia; ES, erector spinae; L3, third lumbar vertebra levels; L4, fourth lumbar vertebra levels; MG, medial gastrocnemius; LG, lateral gastrocnemius.

## Discussion

The purpose of this study was to elucidate the stiffness changes of the lower limb muscle and lumbar tissue during isometric plantar-flexion so as to better understand the interaction between different tissues in the myofascial tensegrity network. This study found that the stiffness changes of the lower limb muscle and lumbar tissue during isometric plantar-flexion had the following characteristics: (1) the stiffness of the TLF, ES, MG, and LG increased with the increase of isometric resistance; (2) there was a strong correlation between the stiffness percentage change of the lumbar tissue and MG; and (3) the stiffness percentage change of the nondominant lumbar tissue was greater than that of the dominant lumbar tissue.

The muscle and fascia tissues do not exist in isolation in structure and function but function together in body movement through a mutual connection forming a myofascial tension network to connect all parts of the body as a whole (Stackhouse et al., 2013)—for example, Barker *et al*. dissected and analyzed the connection structure between the waist, back, and hip and found that TLF is directly connected with the gluteus maximus in the area near the posterior superior iliac spine (PSIS) and S3 spinous process. The aponeurosis of ES extends to be part of the origin of GMax, and the GMax extends downward to be part of the origin of iliotibial tract (Barker et al., 2014). Wilke *et al*. induced a significant displacement of semimembranosus and its fascia band through passive ankle back extension, suggesting that mechanical force can be transmitted from the ankle joint to the dorsal thigh and affect the parallel muscles contained in the dorsal thigh fascia band (biceps femoris and semitendinosus muscle and semimembranosus) during passively stretching the gastrocnemius (Wilke et al., 2020). The anatomical study of Tuncay *et al*. showed that the gastrocnemius and hamstring muscles communicate with each other at the popliteal fossa through the deep fascia of the popliteal fossa (Tuncay et al., 2007). Their results established a possible path for studying the long-distance interaction between the dorsal side of the lower limb muscle and lumbar tissue. The order of the model from distal to proximal is gastrocnemius, deep popliteal fascia, hamstring, dorsal thigh fascia band, GMax, TLF, and ES. In this study, the stiffness of TLF, ES, MG, and LG increased with the increase of plantar isometric bending resistance. The stiffness increase rate of MG was always closely related to the stiffness increase rate of the lumbar tissue (TLF and ES). The mechanical properties of the muscle and fascia depend on the change of their own tension. Tissue stiffness is one of the important indicators to evaluate tissue tension (Swanson, 2013). Therefore, our results preliminarily show that there is a long-distance interaction between the dorsal side of the lower limb muscle and lumbar tissue in the myofascial tensegrity network, and MG, LG, ES, and TLF play a synergistic role in plantar-flexion.

There was no significant difference in lumbar tissue stiffness between the dominant side and the non-dominant side in the resting state, but after the beginning of plantar flexion, the lumbar tissue stiffness of the nondominant side was always greater than that of the dominant side. We speculate that the reason for this difference may be that the plantar flexion movement on the dominant side changes the tension distribution of the lumbar tissues on both sides. Previous studies have shown that passive dorsiflexion of the ankle can induce joint displacement of the gastrocnemius, semimembranosus, and quadriceps femoris (Huang et al., 2018; Wilke et al., 2020). The contraction of Gmax can affect the contralateral latissimus dorsi muscle through TLF and ES (Carvalhais et al., 2013). In an anatomical study, the shallow lamina of TLF crosses the midline at the L4-S2 level and connected with the contralateral sacrum, posterior superior iliac spine, iliac bone, and GMax (Vleeming et al., 1995). Thus, our results reinforce earlier findings of the interaction between different tissues in the myofascial tension network. In addition, as Weisman *et al*. observed, the myoelectric activity of the nondominant side of the PSIS level was significantly higher than that of the dominant side during the maximum resistance plantar-flexion of the dominant foot, indicating that the impulse conduction generated by the lower extremity tissue tension may complete the cross before reaching the PSIS level (Weisman et al., 2014). The studies of Stecco *et al*. have shown that the fascia can sense the tension produced by the contraction of the proximal muscle and transmit the signal to the distance, leading to the activation of the distal muscle (Stecco et al., 2009). Because the fascia has rich proprioceptive innervation and free nerve endings, the stretching of the fascia could activate the corresponding proprioceptors and compress or stretch the free nerve endings (Mense, 2019; Garofolini and Svanera, 2019). Therefore, the interaction between the dorsal side of the lower limb muscle and lumbar tissue may be realized by tension change and nerve signal transmission in myofascial tension network. In the exercise test, the cross-activation mode for the contralateral muscles of the trunk and lower limbs has been proven to be a factor to stabilize the trunk balance (Stevens et al., 2007; García-Vaquero et al., 2012). From the perspective of the whole-body mechanics adjustment, the trunk may have a tendency of dominant flexion when the dominant isometric plantar-flexion occurs. Therefore, the tension structure of the nondominant trunk can increase the tension to fight against the coming shortening of the dominant side and maintain the balance of the body. No matter what the movement mode is, the human trunk always ensures normal function through the overall regulation of the activation mode. This also shows the importance of studying the whole-body mechanics regulation mode, and the study of the myofascial tensegrity network is just one of these links.

Plantar-flexion is an important part of walking, running, jumping, and other activities. ln the process of the foot pushing off the ground, it is accompanied by isometric plantar-flexion and a continuous increase of plantar-flexion myodynamia (Li et al., 2018). Therefore, studying the effect of isometric plantar-flexion on tissue stiffness can clarify the adjustment strategy of different tissues in isometric plantar-flexion. After analyzing the stiffness-phased percentage change of various tissues at different resistances, we found that the stiffness-phased percentage change of ES and MG is much higher than that of TLF and LG when the resistance changed from 0 to 20%MVIC, which indicates that MG is the main generation part of plantar-flexion tension, and ES is the lumbar tissue most affected by the change of lower limb tension at the initial stage of resistance rising. When the resistance changed from 20 to 40%MVIC, the stiffness-phased percentage change of LG increased significantly and that of MG decrease significantly. We speculate that, when the resistance changes from 20 to 40%, the output strategies of MG and LG are redistributed, the force output of MG tends to be stable, and LG improves the strength of the force output. Because MG and LG are laterally adjacent and converge on the Achilles tendon, the lateral disturbance of adjacent muscles and tension transmission between tendons may be the reason for this phenomenon, which needs further research to verify (Huijing and Baan, 2001). Moreover, when the resistance reaches 60% MVIC, the stiffness-phased percentage change of TLF, ES, MG, and LG decreased significantly, indicating that the stiffness changes of each tissue tended to be stable.

## Limitations

There are some limitations to this study. First, we failed to measure the thigh portion of the myofascial tensegrity network due to technical reasons. Second, the ankle 90° fixator covers the distal end of the soleus muscle and the Achilles tendon, so we could not evaluate these two areas. Third, only female participants were recruited. In our preliminary experiments, the tissue stiffness of 40 and 60% MVIC in male participants often exceeded the measuring range of the instrument. Therefore, only female individuals were recruited in this study. We need to develop instruments with a larger measuring range to meet the needs of future research. Fourth, although we fully trained the participants to not contract the auxiliary muscles, due to the lack of EMG monitoring equipment, we cannot confirm that this was the case. In the next experiment, we will introduce an EMG device to objectively monitor the muscle activity. Finally, the results of this study were only applicable to healthy young people. We will gradually carry out research on injured people in the future.

## Conclusion

This study quantified stiffness values of the TLF, ES, MG, and LG in healthy humans during isometric plantar-flexion against different resistances using the SWE. We found a moderate to strong correlation between TLF, ES, MG, and LG during isometric plantar-flexion, which provides preliminary data for exploring the interaction between different tissues in the myofascial tensegrity network. Through the study of different resistance conditions, we know that the change of local force will have a synergistic effect on other parts of the myofascial tensegrity network, which provides a valuable reference for us to understand the overall regulation mode of the myofascial tensegrity network of injury more comprehensively.

## Data Availability

The raw data supporting the conclusion of this article will be made available by the authors without undue reservation.
