# Supplementary material for: Effects of Isometric Plantar-Flexion on the Lower Limb Muscle and Lumbar Tissue Stiffness
Source: Front Bioeng Biotechnol. 2022 Feb 11;9:810250. doi: 10.3389/fbioe.2021.810250 (PMC8874132; doi:10.3389/fbioe.2021.810250)
Supplement: Supplementary file 2 [file Table1.docx]

| Supplementary Table 1. ANOVA results for tissue stiffness | | | | | | | | |
| --- | --- | --- | --- | --- | --- | --- | --- | --- |
| Independent variables | Position | | | Degrees of Freedom | Sum of Squares | Mean Square | F | P |
| Resistance  (0%MVIC, 20%MVIC, 40%MVIC, 60%MVIC) | TLF | L3 | Left | 3 | 254914.815 | 84971.605 | 2111.445 | 0.000 |
|  |  |  | Right | 3 | 103469.742 | 34489.914 | 266.536 | 0.000 |
|  |  | L4 | Left | 3 | 253038.403 | 84346.134 | 1650.907 | 0.000 |
|  |  |  | Right | 3 | 94603.793 | 31534.598 | 811.689 | 0.000 |
|  | ES | L3 | Left | 3 | 328927.403 | 109642.468 | 2334.395 | 0.000 |
|  |  |  | Right | 3 | 216429.972 | 72143.324 | 993.144 | 0.000 |
|  |  | L4 | Left | 3 | 317040.587 | 105680.196 | 999.726 | 0.000 |
|  |  |  | Right | 3 | 237428.994 | 79142.998 | 765.103 | 0.000 |
|  | MG | | | 3 | 208370.003 | 69456.668 | 422.449 | 0.000 |
|  | LG | | | 3 | 128788.058 | 42929.353 | 751.330 | 0.000 |
