# Supplementary figures and images for: Effects of Isometric Plantar-Flexion on the Lower Limb Muscle and Lumbar Tissue Stiffness
Source: Front Bioeng Biotechnol. 2022 Feb 11;9:810250. doi: 10.3389/fbioe.2021.810250 (PMC8874132; doi:10.3389/fbioe.2021.810250)

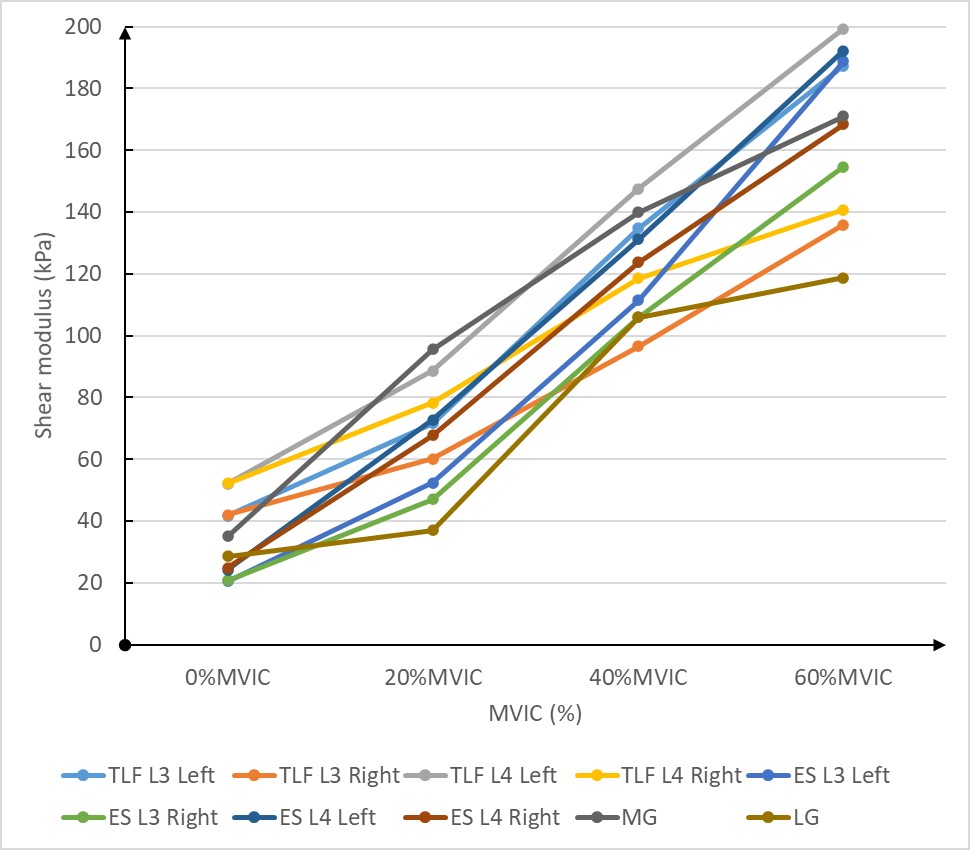

Supplement: Supplementary file 4 [file Image4.JPEG]
