# Supplementary material for: Effects of Isometric Plantar-Flexion on the Lower Limb Muscle and Lumbar Tissue Stiffness
Source: Front Bioeng Biotechnol. 2022 Feb 11;9:810250. doi: 10.3389/fbioe.2021.810250 (PMC8874132; doi:10.3389/fbioe.2021.810250)
Supplement: Supplementary file 6 [file Table2.docx]

| Supplementary Table 2. Post-hoc comparisons results for tissue stiffness | | | | | | | |
| --- | --- | --- | --- | --- | --- | --- | --- |
| Position | | | (I)MVIC | (J)MVIC | Mean Difference(I-J) | Std.Error | p |
| TLF | L3 | Left | 0%MVIC | 20%MVIC | -29.969 | 2.006 | 0.000 |
|  |  |  |  | 40%MVIC | -93.056 | 2.006 | 0.000 |
|  |  |  |  | 60%MVIC | -145.783 | 2.006 | 0.000 |
|  |  |  | 20%MVIC | 40%MVIC | -63.087 | 2.006 | 0.000 |
|  |  |  |  | 60%MVIC | -115.814 | 2.006 | 0.000 |
|  |  |  | 40%MVIC | 60%MVIC | -52.727 | 2.006 | 0.000 |
|  |  | Right | 0%MVIC | 20%MVIC | -18.081 | 3.597 | 0.000 |
|  |  |  |  | 40%MVIC | -54.430 | 3.597 | 0.000 |
|  |  |  |  | 60%MVIC | -93.803 | 3.597 | 0.000 |
|  |  |  | 20%MVIC | 40%MVIC | -36.350 | 3.597 | 0.000 |
|  |  |  |  | 60%MVIC | -75.723 | 3.597 | 0.000 |
|  |  |  | 40%MVIC | 60%MVIC | -39.373 | 3.597 | 0.000 |
|  | L4 | Left | 0%MVIC | 20%MVIC | -36.554 | 2.260 | 0.000 |
|  |  |  |  | 40%MVIC | -95.505 | 2.260 | 0.000 |
|  |  |  |  | 60%MVIC | -147.349 | 2.260 | 0.000 |
|  |  |  | 20%MVIC | 40%MVIC | -58.951 | 2.260 | 0.000 |
|  |  |  |  | 60%MVIC | -110.790 | 2.260 | 0.000 |
|  |  |  | 40%MVIC | 60%MVIC | -51.844 | 2.260 | 0.000 |
|  |  | Right | 0%MVIC | 20%MVIC | -26.070 | 1.971 | 0.000 |
|  |  |  |  | 40%MVIC | -66.347 | 1.971 | 0.000 |
|  |  |  |  | 60%MVIC | -88.490 | 1.971 | 0.000 |
|  |  |  | 20%MVIC | 40%MVIC | -40.278 | 1.971 | 0.000 |
|  |  |  |  | 60%MVIC | -62.420 | 1.971 | 0.000 |
|  |  |  | 40%MVIC | 60%MVIC | -22.143 | 1.971 | 0.000 |
| ES | L3 | Left | 0%MVIC | 20%MVIC | -31.908 | 2.167 | 0.000 |
|  |  |  |  | 40%MVIC | -91.004 | 2.167 | 0.000 |
|  |  |  |  | 60%MVIC | -168.419 | 2.167 | 0.000 |
|  |  |  | 20%MVIC | 40%MVIC | -59.096 | 2.167 | 0.000 |
|  |  |  |  | 60%MVIC | -136.511 | 2.167 | 0.000 |
|  |  |  | 40%MVIC | 60%MVIC | -77.415 | 2.167 | 0.000 |
|  |  | Right | 0%MVIC | 20%MVIC | -26.206 | 2.695 | 0.000 |
|  |  |  |  | 40%MVIC | -85.181 | 2.695 | 0.000 |
|  |  |  |  | 60%MVIC | -133.839 | 2.695 | 0.000 |
|  |  |  | 20%MVIC | 40%MVIC | -58.975 | 2.695 | 0.000 |
|  |  |  |  | 60%MVIC | -107.634 | 2.695 | 0.000 |
|  |  |  | 40%MVIC | 60%MVIC | -48.659 | 2.695 | 0.000 |
|  | L4 | Left | 0%MVIC | 20%MVIC | -48.673 | 3.251 | 0.000 |
|  |  |  |  | 40%MVIC | -106.980 | 3.251 | 0.000 |
|  |  |  |  | 60%MVIC | -168.012 | 3.251 | 0.000 |
|  |  |  | 20%MVIC | 40%MVIC | -58.307 | 3.251 | 0.000 |
|  |  |  |  | 60%MVIC | -119.339 | 3.251 | 0.000 |
|  |  |  | 40%MVIC | 60%MVIC | -61.033 | 3.251 | 0.000 |
|  |  | Right | 0%MVIC | 20%MVIC | -42.836 | 3.216 | 0.000 |
|  |  |  |  | 40%MVIC | -98.934 | 3.216 | 0.000 |
|  |  |  |  | 60%MVIC | -143.508 | 3.216 | 0.000 |
|  |  |  | 20%MVIC | 40%MVIC | -56.098 | 3.216 | 0.000 |
|  |  |  |  | 60%MVIC | -100.672 | 3.216 | 0.000 |
|  |  |  | 40%MVIC | 60%MVIC | -44.574 | 3.216 | 0.000 |
| MG | | | 0%MVIC | 20%MVIC | -60.514 | 4.055 | 0.000 |
|  |  |  |  | 40%MVIC | -104.753 | 4.055 | 0.000 |
|  |  |  |  | 60%MVIC | -135.822 | 4.055 | 0.000 |
|  |  |  | 20%MVIC | 40%MVIC | -44.229 | 4.055 | 0.000 |
|  |  |  |  | 60%MVIC | -75.309 | 4.055 | 0.000 |
|  |  |  | 40%MVIC | 60%MVIC | -31.080 | 4.055 | 0.000 |
| LG | | | 0%MVIC | 20%MVIC | -8.518 | 2.390 | 0.000 |
|  |  |  |  | 40%MVIC | -77.417 | 2.390 | 0.000 |
|  |  |  |  | 60%MVIC | -90.127 | 2.390 | 0.000 |
|  |  |  | 20%MVIC | 40%MVIC | -68.900 | 2.390 | 0.000 |
|  |  |  |  | 60%MVIC | -81.610 | 2.390 | 0.000 |
|  |  |  | 40%MVIC | 60%MVIC | -12.710 | 2.390 | 0.000 |
