# Supplementary material for: Effects of Isometric Plantar-Flexion on the Lower Limb Muscle and Lumbar Tissue Stiffness
Source: Front Bioeng Biotechnol. 2022 Feb 11;9:810250. doi: 10.3389/fbioe.2021.810250 (PMC8874132; doi:10.3389/fbioe.2021.810250)
Supplement: Supplementary file 7 [file DataSheet1.PDF]

Supplementary Material

Supplementary Figure 1.: Shear wave elastic ultrasound image of erector spinae. (A: 0%MVIC-Left; B: 0%MVIC-Right; C:20%MVIC-Left; D: 20%MVIC-Right; E: 40%MVIC-Left; F: 40%MVIC-Right; G: 60%MVIC-Left; F: 60%MVIC-Right)

Supplementary Figure 2.: Shear wave elastic ultrasound images of medial gastrocnemius (MG) and lateral gastrocnemius (LG). (A: 0%MVIC-MG; B: 20%MVIC-MG; C:40%MVIC-MG D: 60%MVIC-MG; E: 0%MVIC-LG; F: 20%MVIC-LG; G: 40%MVIC-LG; F: 60%MVIC-LG)

Supplementary Figure 3.: Shear wave elastic ultrasound image of thoracolumbar fascia. (A: 0%MVIC-Left; B: 0%MVIC-Right; C:20%MVIC-Left; D: 20%MVIC-Right; E: 40%MVIC-Left; F: 40%MVIC-Right; G: 60%MVIC-Left; F: 60%MVIC-Right)

Supplementary Figure 4.: Effects of isometric plantar-flexion on the lower limb muscle and lumbar tissue stiffness.
